# Volume change after maxillary sinus floor elevation with apatite carbonate and octacalcium phosphate

**DOI:** 10.1186/s40729-023-00518-7

**Published:** 2024-02-08

**Authors:** Koudai Nagata, Masanobu Kamata, Yurie Okuhama, Kana Wakamori, Manabu Okubo, Hayoto Tsuruoka, Mihoko Atsumi, Hiromasa Kawana

**Affiliations:** 1 Department of Oral and Maxillofacial Implantology, Kanagawa Dental University, 82 Inaoka-Cho, Yokosuka, 238-8580 Japan; 2 Department of Fixed Prosthodontics, Kanagawa Dental University, 82 Inaoka-Cho, Yokosuka, 238-8580 Japan

**Keywords:** Dental implant, Maxillary sinus floor elevation, Bone substitute material, Carbonate apatite, Octacalcium phosphate

## Abstract

**Purpose:**

Maxillary molars have low alveolar bone height diameter due to the presence of the maxillary sinus; thus, a sinus lift may be required in some cases. Changes in the volume of bone substitutes can affect the success of implant therapy. Therefore, this study aimed to compare the changes in the volume of two different bone substitutes—one based on carbonate apatite and the other on octacalcium phosphate—used in maxillary sinus floor elevation.

**Methods:**

Nineteen patients and 20 sites requiring maxillary sinus floor elevation were included in the study. Digital Imaging and Communications in Medicine data for each patient obtained preoperatively and immediately and 6 months postoperatively were used to measure the volume of the bone grafting material using a three-dimensional image analysis software. The immediate postoperative volume of octacalcium phosphate was 95.3775 mm^3^ per piece of grafting material used. It was multiplied by the number of pieces used and converted to mL to determine the immediate postoperative volume.

**Results:**

The mean resorption values of carbonate apatite and octacalcium phosphate were 12.7 ± 3.6% and 17.3 ± 3.9%, respectively. A significant difference in the amount of resorption of the two bone replacement materials was observed (*P* = 0.04).

**Conclusions:**

The results of this study indicate that both bone substitute materials tend to resorb. The two bone grafting materials that are currently medically approved in Japan have not been in the market for a long time, and their long-term prognosis has not yet been reported. Further clinical data are warranted.

## Background

Implant treatment using materials with various surface properties and implant morphologies has recently become available, enabling the provision of treatment tailored to the needs of the patient, such as shorter treatment times and immediate loading [1–3]. However, the alveolar bone height in the posterior maxilla may be low due to presence of the maxillary sinus. The alveolar bone height may decrease after the loss of molars, thereby warranting a sinus floor augmentation procedure in certain cases that require implant rehabilitation. Although the survival rate of short implants placed in the posterior region of the maxilla was not significantly different than that of standard-length implants, the long-term prognosis is unknown [4]. Lin et al. [5] reported an average alveolar bone height of 6.62 ± 1.32 mm in maxillary molars before extraction. However, bone resorption commonly occurs horizontally and vertically after tooth extraction [6]. Therefore, even with short implants, implant treatment may not be possible without the use of techniques such as maxillary sinus floor elevation [7, 8]. Various types of bone substitutes are used for maxillary sinus floor elevation, and selecting the appropriate material is important in order to obtain long-term survival rates for implant treatment [9–11]. As a bone substitute, only autogenous bone has osteogenesis, osteoinduction, and osteoconduction capabilities. However, because of the invasive nature of the procedure and limited amount of bone that can be harvested, artificial materials are increasingly used [12]. In Japan, Cytrans®, a granular formulation based on carbonated apatite, was launched in 2018 with the first medical approval for an implant indication [13]. In 2022, Bonarc®, also medically approved for implant indications, was launched in sponge form and composed mainly of octacalcium phosphate (OCP) and collagen (Col) (80 wt% OCP and 20 wt% Col) [14]. Because these two materials have only been on the market for a short time, there are few clinical reports and no studies comparing the changes in volume of the two materials. Changes in the volume of bone substitute after maxillary sinus floor elevation can affect the prognosis of implant therapy. In measuring changes in the volume of bone substitute after maxillary sinus floor elevation, Digital Imaging and Communications in Medicine (DICOM) data after preoperative and postoperative computed tomography (CT) imaging are usually measured using three-dimensional image analysis software [15, 16]. Therefore, this study aimed to compare the volume changes of Cytrans® and Bonarc® as substitutes for sinus lifts immediately after surgery using a three-dimensional image analysis software.

## Methods

**Fig. 1:**
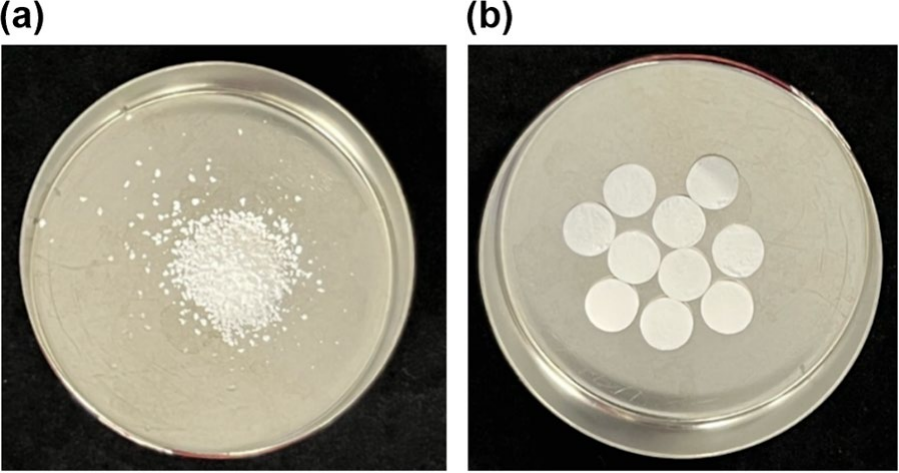
Bone substitute material used. **a** Cytrans® (size, M; particle size, 0.6–1.0 mm). **b** Bonarc® (disk)

The study included 19 patients (10 males and 9 females; 20 sites) with a mean age of 59.1 years, who required maxillary sinus floor elevation for implant treatment. Nine patients and 10 sites were included in the Cytrans® (GC, Tokyo, Japan) group, and 10 patients and 10 sites were included in the Bonarc® (TOYOBO CO., LTD., Shiga, Japan) group. Patients had to be at least 20 years old, non-smokers, free of systemic disease, and have no thickening of the maxillary sinus mucosa on cone-beam CT (CBCT) imaging. All maxillary sinus floor elevations were performed with a staged approach using the lateral window technique [17]. Either Cytrans® (size, M; particle size, 0.6–1.0 mm) or Bonarc® (disk) was used as a bone substitute material for maxillary sinus floor elevation (Fig. 1). These procedures were conducted once the study parameters were explained to the patients, and their consent was obtained. This study was approved by the Kanagawa Dental University Ethics Committee (approval number: 906).

### Volume measurement

The volume of bone substitute material was determined using CBCT images (3DX®, Morita, Tokyo, Japan), with T1 set for preoperative, T2 for immediate postoperative, and T3 for 6 months postoperative. During the imaging, the upper and lower dentition were opened such that they did not overlap. The obtained DICOM data of each patient was then used to measure the volume using a three-dimensional image analysis software (SYNAPSE VINCENT®, FUJIFILM, Tokyo, Japan). In the Cytarans® group, the fusion function of SYNAPSE VINCENT® was used to superimpose T1 and T2 data to calculate T2 immediately after surgery, and T1 and T3 data to calculate T3, the volume 6 months postoperative. Bonarc® has low X-ray permeability, which makes identification of the enlarged volume after CBCT imaging challenging. Since Bonarc® (disk) has a diameter of 9 mm and a height of 1.5 mm, the volume of the cylinder was determined to be 4.5 mm × 4.5 mm × 3.14 × 1.5 mm, or 95.3775 mm^3^ per piece. The volume of T2 was measured by multiplying the volume of this single sheet by the number of sheets used and converting the units to mL. The volume of T3 was measured by superimposing the T1 and T3 data as in the Cytarans® group. In both cases, the change in volume was measured by subtracting T2 from T3.

**Fig. 2:**
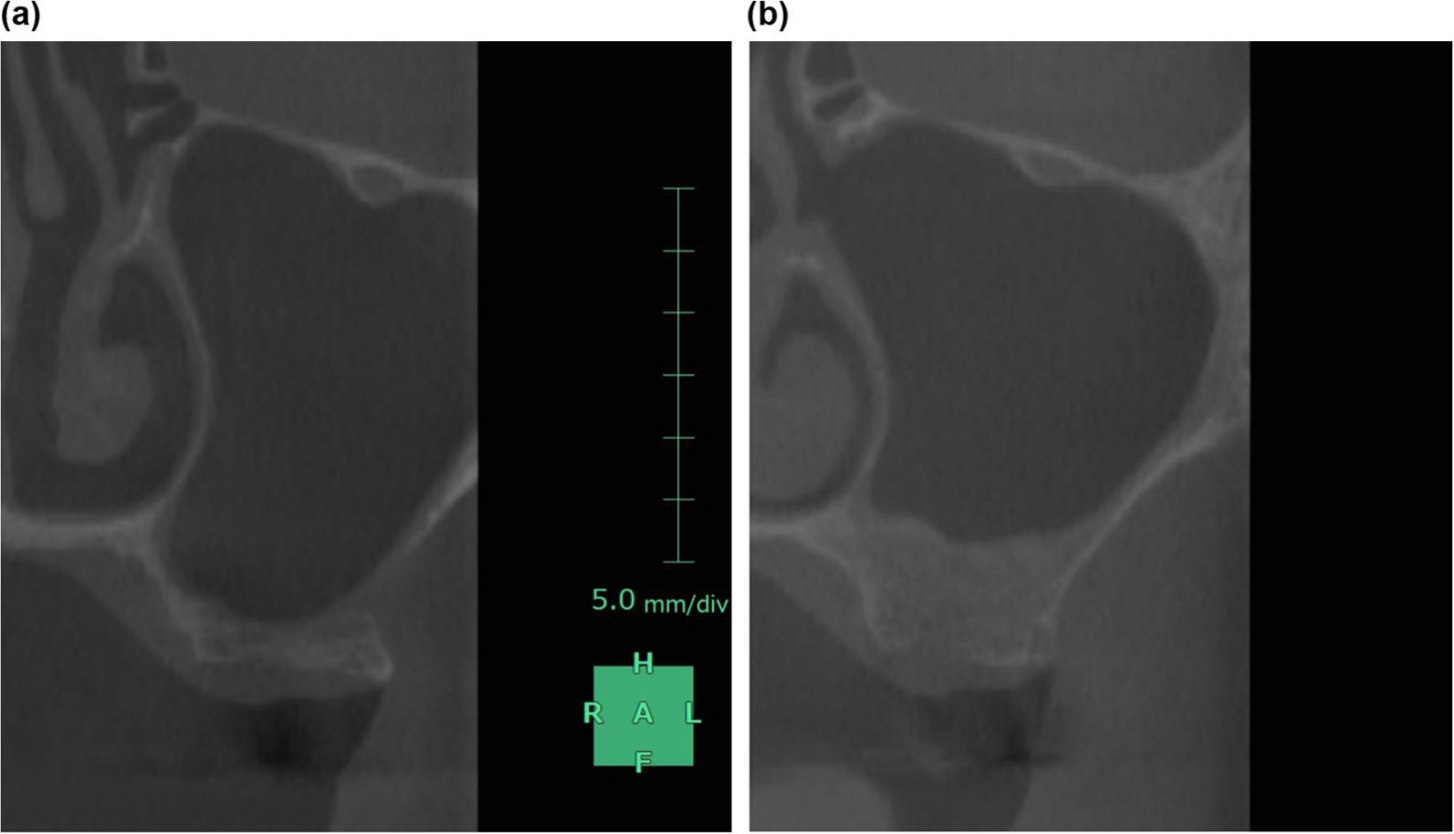
**a** Preoperative cone-beam computed tomography (CBCT) data. **b** Postoperative CBCT data

**Fig. 3:**
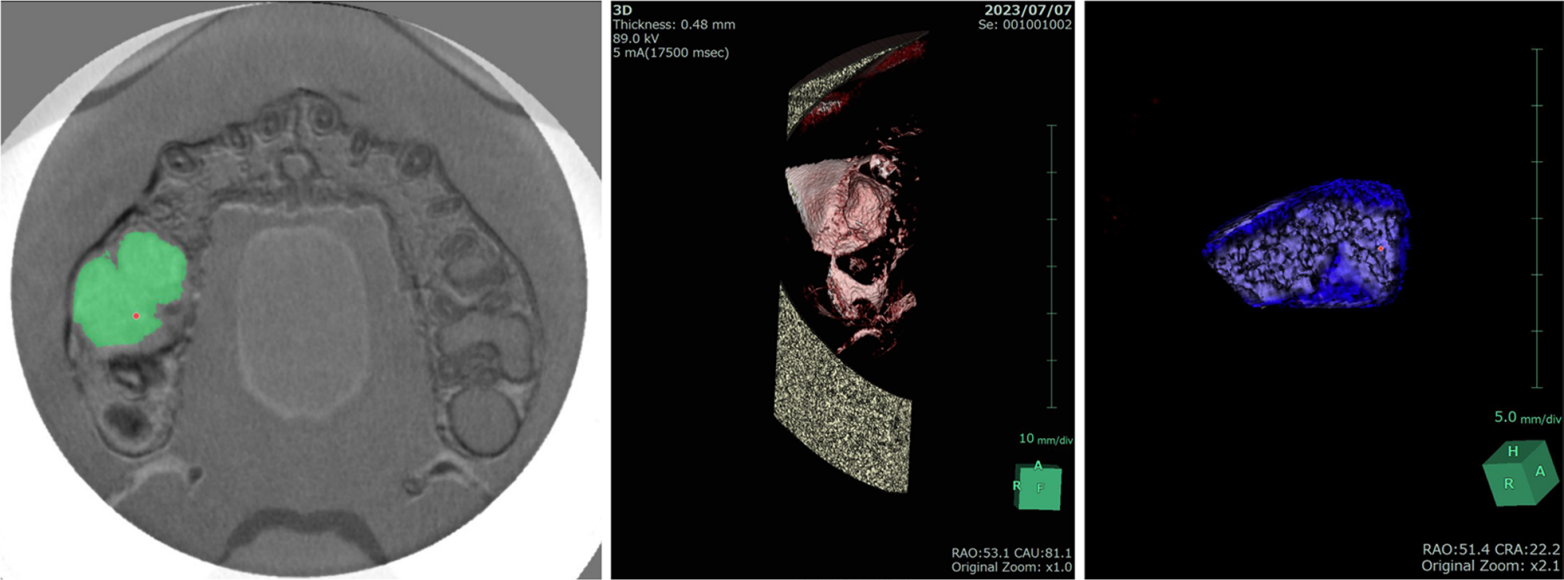
The excess data were trimmed, and the volume of bone replacement material was measured

The SYNAPSE VINCENT® measurement method was based on the “manual image alignment” option of the fusion function, which was selected by importing preoperative and postoperative DICOM, and superimposed with reference to the remaining dentition (Fig. 2). Afterwards, “image reconstruction” was selected, excess data were trimmed from the obtained 3D data, and “overall measurement” was selected to measure the volume of the remaining bone. The volume of the remaining bone substitute was measured [18] (Fig. 3).

### Statistical analysis

The Student’s *t*-test (*P* < 0.05) was used to compare the amount of resorption of each bone substitute material. The statistical analysis software used was BellCurve for Excel (Social Survey Research Information Co., Ltd., Tokyo, Japan). The sample size was not calculated because of the limited number of cases in this study.

## Results

**Fig. 4:**
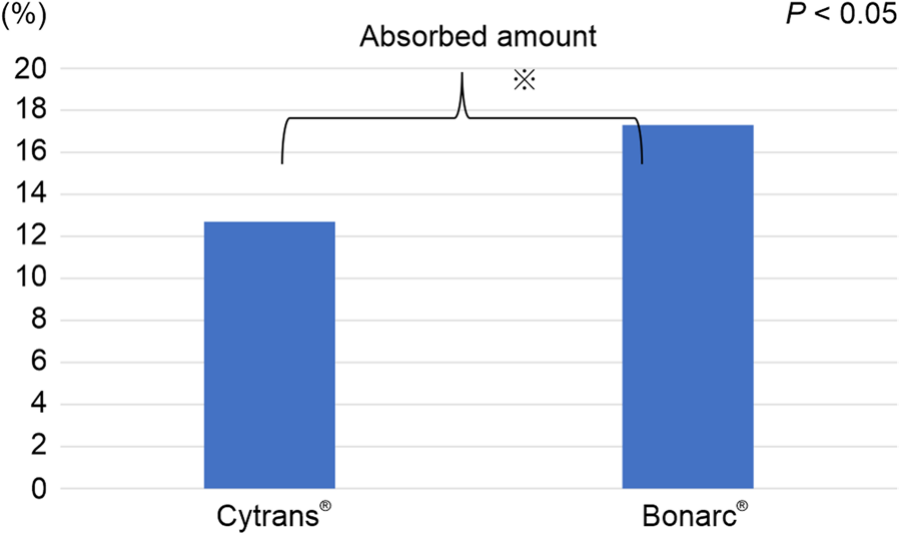
Amount of resorption of Cytrans® and Bonarc®

The results of this study showed that Cytrans® resorbed an average of 12.7 ± 3.6% and Bonarc® resorbed an average of 17.3 ± 3.9%. Significant differences in the resorption of the two bone substitutes were observed (*P* = 0.04) (Fig. 4). The respective resorption amounts are shown in Table 1.

**Table 1:** Clinical data on the volume of bone-filling material in procedures using Cytrans® and Bonarc®

Cytrans®	Age	Sex	Deficit condition (FDI)	T2 (mL)	T3 (mL)	Absorbed amount (%)	
1	67	F	14–17	2.547	2.371	6.90	
2	41	F	16	2.108	1.791	15.00	
3	59	M	26,27	1.506	1.222	18.90	
4	67	M	Edentulous	3.763	3.375	10.30	
5	67	M	Edentulous	2.392	2.15	10.10	
6	67	F	24–27	2.78	2.386	14.20	
7	56	F	16,17	2.543	2.241	10.90	
8	62	M	15,16	2.863	2.568	10.30	
9	56	M	15,16	3.237	3.085	15.20	
10	70	M	15,16	1.42	1.261	15.90	

Bonarc®	Age	Sex	Deficit condition (FDI)	Number of sheets	T2 (mL)	T3 (mL)	Absorbed amount (%)
1	40	M	26	16	1526.0	1323	13.30
2	69	M	16	10	953.8	724.3	24.10
3	80	F	13–16	13	1239.9	1068	13.90
4	21	M	14–16	17	1621.4	1250	22.90
5	61	F	26	20	1907.6	1596	16.30
6	67	F	25–27	18	1716.8	1474	14.10
7	73	F	24–27	20	1907.6	1613.2	15.40
8	40	M	24–27	15	1430.7	1139.3	20.40
9	59	M	24–27	20	1907.6	1628.4	14.60
10	68	F	14–17	20	1907.6	1563	18.10

## Discussion

The results of this study showed a significant difference in the amount of resorption of the two bone substitutes. Carbonate apatite has the same composition as hydroxyapatite and the inorganic component of bone. It has been clinically applied for many years, and its safety has been confirmed; thus, it is used as a substitute for maxillary sinus floor elevation and socket preservation [19, 20].

Kudoh et al. [13] performed maxillary sinus floor elevation in eight patients using carbonate apatite. They reported that the postoperative bone height diameter increased to 14.0 ± 1.9 mm but decreased to 12.4 ± 1.3 mm after 7 months and to 11.7 ± 0.6 mm after 12 months after prosthetic loading. Nakagawa et al. [21] also reported that maxillary sinus floor elevation using carbonate apatite in 13 patients increased to 13.3 ± 1.7 mm postoperatively and decreased to 9.6 ± 1.4 mm after 18 months. The results were similar to ours such that carbonate apatite tended to resorb after maxillary sinus floor elevation. Mano et al. [22] measured the amount of new bone formation of NEOBONE, Bio-Oss, and Cytrans containing 0.1, 5.5, and 12.0% carbonate, respectively, in dogs. It was reported that NEOBONE had 4.7%, Bio-Oss 39.5%, and Cytrans 75.2% of new bone at 12 weeks postoperatively, with Cytrans inducing the highest amount of new bone. High carbonate content reportedly plays an important role in bone replacement and high osteoconductivity [23]. Therefore, Cytrans induced a higher amount of new bone mass. Atsuta et al. [24] reported that mixing carbonated apatite with autologous bone, rather than carbonated apatite alone, increases osteoclasts and osteoblasts and provides additional osteogenesis. This may lead to shorter sinus lift treatment time and further reduction of resorption. The prognosis of maxillary sinus floor elevation with carbonate apatite is not encouraging; Ogino et al. [25] reported a 100% survival rate at 3 years after 17 implantations in 13 patients, and long-term prognosis is expected in the future.

OCP is considered a precursor of biological apatite crystals in bones and teeth. It has been proven to promote osteoblast differentiation and bone regeneration and is indicated for medical treatment [26, 27]. Kawai et al. [28] used OCP/Col for the first time in human bone defects and reported that postoperative wound healing was uneventful, and no infection or allergic reaction was observed. Miura et al. [14] were the first to use OCP/Col as a substitute for maxillary sinus floor elevation in clinical practice. They reported that the vertical bone height diameters of six sites in a staged approach averaged at 15.8 mm at 3 months, 14.4 mm at 6 months, and 14.3 mm at 12 months after surgery. Miura et al. [29] also reported a change in volume in three patients who underwent maxillary sinus floor elevation using OCP/Col: 4.79 cm^3^ at 6 months versus 4.61 cm^3^ at 1 year. There were no reports that measured the volume of OCP/Col immediately after maxillary sinus floor elevation. Therefore, this study is the first report to measure volume absorption immediately after surgery. We also considered the possibility that Bonarc® might increase in volume when used for maxillary sinus floor elevation. However, considering this study and these previous reports, there is clearly a trend toward resorption immediately after the procedure and over time, similar to that in carbonate apatite. Because Bonarc® had only been on the market for a short time, no prognostic reports existed. The results of this study showed that Bonarc® resulted in greater absorption. Although Bonarc® is spongy and therefore, easier to manipulate, we believe that the amount of resorption was greater than that of the granular bone substitute because of the gaps between the materials. Because Bonarc® has low X-ray permeability, measuring the volume immediately after surgery from DICOM is challenging; thus, the overall volume was calculated from the volume per piece. On the other hand, the granularity of Cytrans® makes it difficult to accurately measure the volume used intraoperatively. Therefore, T2 for the two materials were measured in different ways. Since both T2 and T3 of Cytrans® measure volume from DICOM superimposition, Cytrans® may have a larger error margin when artifact issues are taken into account. The densities at T3 of the two materials may also be different. Although obtaining CT values would be ideal to reduce errors in volumetric measurements, it was not possible the present study as CBCT was used. This issue should be addressed in future studies.

Regarding insertion torque, Kawai et al. [30] performed maxillary sinus floor elevation in a staged approach using OCP/Col. The insertion torque values were less than 20 Ncm in 25 patients, and they reported that this was due to the fact that the majority of the bone was newly formed and there was little mature bone. On the other hand, Ogino et al. [25] reported an average insertion torque of 25.1 ± 13.2 Ncm for 17 implants after maxillary sinus floor elevation using carbonated apatite. However, insertion torque depending on the implant system used, bone quality, alveolar bone height diameter, and technique is still not considered; thus, more detailed data are needed in the future. Our study had certain limitations. First, the sample size was small owing to the limited number of cases of maxillary sinus floor elevation. Second, the prognosis after implantation was not tracked in the present study. We plan to report on the system, initial fixation, and survival rates in future studies. Only two bone substitutes, Cytrans® and Bonarc®, are currently indicated for implantation. Moreover, due to the paucity of clinical reports, future reports on their long-term prognosis are needed.

## Conclusions

Two bone substitutes currently approved for medical use were found to have a tendency to resorb after maxillary sinus floor elevation. A significant difference in the amount of resorption was also observed. Short-term prognosis has been reported for Cytrans®, which is slightly earlier in the market, but not for Bonara®. Moreover, long-term prognosis for both is still to be reported.

## Data Availability

The datasets obtained and analyzed during the current study are available from the corresponding author upon reasonable request.

## Abbreviations

OCP: Octacalcium phosphate
Col: Collagen
CBCT: Cone-beam computed tomography
DICOM: Digital Imaging and Communications in Medicine

## References

[1] Chambrone L, Shibli JA, Mercúrio CE, Cardoso B, Preshaw PM (2015). Efficacy of standard (SLA) and modified sandblasted and acid-etched (SLActive) dental implants in promoting immediate and/or early occlusal loading protocols: a systematic review of prospective studies. Clin Oral Implants Res.

[2] Moretto D, Gargari M, Nordsjö E, Gloria F, Ottria L (2008). Immediate loading: a new implant technique with immediate loading and aesthetics: Nobel Active™. Oral Implantol.

[3] Papaspyridakos P, De Souza A, Vazouras K, Gholami H, Pagni S, Weber HP (2018). Survival rates of short dental implants (≤6 mm) compared with implants longer than 6 mm in posterior jaw areas: a meta-analysis. Clin Oral Implants Res.

[4] Carosi P, Lorenzi C, Lio F, Laureti M, Ferrigno N, Arcuri C (2021). Short implants (≤6 mm) as an alternative treatment option to maxillary sinus lift. Int J Oral Maxillofac Surg.

[5] Lin HK, Pan YH, Salamanca E, Lin YT, Chang WJ (2019). Prevention of bone resorption by HA/β-TCP + collagen composite after tooth extraction: a case series. Int J Environ Res Public Health.

[6] Tan WL, Wong TL, Wong MC, Lang NP (2012). A systematic review of post-extractional alveolar hard and soft tissue dimensional changes in humans. Clin Oral Implants Res.

[7] Walter C, Dagassan-Berndt DC, Kühl S, Weiger R, Lang NP, Zitzmann NU (2014). Is furcation involvement in maxillary molars a predictor for subsequent bone augmentation prior to implant placement? A pilot study. Clin Oral Implants Res.

[8] Tian XM, Qian L, Xin XZ, Wei B, Gong Y (2016). An analysis of the proximity of maxillary posterior teeth to the maxillary sinus using cone-beam computed tomography. J Endod.

[9] Sheikh Z, Sima C, Glogauer M (2015). Bone replacement materials and techniques used for achieving vertical alveolar bone augmentation. Materials.

[10] Starch-Jensen T, Jensen JD (2017). Maxillary sinus floor augmentation: a review of selected treatment modalities. J Oral Maxillofac Res.

[11] Mordenfeld A, Lindgren C, Hallman M (2016). Sinus floor augmentation using Straumann® BoneCeramic™ and Bio-Oss® in a split mouth design and later placement of implants: a 5-year report from a longitudinal study. Clin Implant Dent Relat Res.

[12] Sato R, Matsuura T, Akizuki T, Fukuba S, Okada M, Nohara K (2022). Influence of the bone graft materials used for guided bone regeneration on subsequent peri-implant inflammation: an experimental ligature-induced peri-implantitis model in Beagle dogs. Int J Implant Dent.

[13] Kudoh K, Fukuda N, Kasugai S, Tachikawa N, Koyano K, Matsushita Y (2019). Maxillary sinus floor augmentation using low-crystalline carbonate apatite granules with simultaneous implant installation: first-in-human clinical trial. J Oral Maxillofac Surg.

[14] Miura KI, Sumita Y, Kajii F, Tanaka H, Kamakura S, Asahina I (2020). First clinical application of octacalcium phosphate collagen composite on bone regeneration in maxillary sinus floor augmentation: a prospective, single-arm, open-label clinical trial. J Biomed Mater Res B Appl Biomater.

[15] Kwon JJ, Hwang J, Kim YD, Shin SH, Cho BH, Lee JY (2019). Automatic three-dimensional analysis of bone volume and quality change after maxillary sinus augmentation. Clin Implant Dent Relat Res.

[16] Pichotano EC, de Molon RS, de Souza RV, Austin RS, Marcantonio E, Zandim-Barcelos DL (2019). Evaluation of L-PRF combined with deproteinized bovine bone mineral for early implant placement after maxillary sinus augmentation: a randomized clinical trial. Clin Implant Dent Relat Res.

[17] Boyne PJ, James RA (1980). Grafting of the maxillary sinus floor with autogenous marrow and bone. J Oral Surg.

[18] Nagata K, Fuchigami K, Kitami R, Okuhama Y, Wakamori K, Sumitomo H (2021). Comparison of the performances of low-crystalline carbonate apatite and Bio-Oss in sinus augmentation using three-dimensional image analysis. Int J Implant Dent.

[19] Spence G, Patel N, Brooks R, Bonfield W, Rushton N (2010). Osteoclastogenesis on hydroxyapatite ceramics: the effect of carbonate substitution. J Biomed Mater Res A.

[20] Egashira Y, Atsuta I, Narimatsu I, Zhang X, Takahashi R, Koyano K (2022). Effect of carbonate apatite as a bone substitute on oral mucosal healing in a rat extraction socket: in vitro and in vivo analyses using carbonate apatite. Int J Implant Dent.

[21] Nakagawa T, Kudoh K, Fukuda N, Kasugai S, Tachikawa N, Koyano K (2019). Application of low-crystalline carbonate apatite granules in 2-stage sinus floor augmentation: a prospective clinical trial and histomorphometric evaluation. J Periodontal Implant Sci.

[22] Mano T, Akita K, Fukuda N, Kamada K, Kurio N, Ishikawa K (2020). Histological comparison of three apatitic bone substitutes with different carbonate contents in alveolar bone defects in a beagle mandible with simultaneous implant installation. J Biomed Mater Res B Appl Biomater.

[23] Fujisawa K, Akita K, Fukuda N, Kamada K, Kudoh T, Ohe G (2018). Compositional and histological comparison of carbonate apatite fabricated by dissolution-precipitation reaction and Bio-Oss®. J Mater Sci Mater Med.

[24] Atsuta I, Mizokami T, Jinno Y, Ji B, Xie T, Ayukawa Y (2022). Synergistic effect of carbonate apatite and autogenous bone on osteogenesis. Materials.

[25] Ogino Y, Ayukawa Y, Tachikawa N, Shimogishi M, Miyamoto Y, Kudoh K (2021). Staged sinus floor elevation using novel low-crystalline carbonate apatite granules: prospective results after 3-year functional loading. Materials.

[26] Anada T, Kumagai T, Honda Y, Masuda T, Kamijo R, Kamakura S (2008). Dose-dependent osteogenic effect of octacalcium phosphate on mouse bone marrow stromal cells. Tissue Eng Part A.

[27] Suzuki O, Kamakura S, Katagiri T, Nakamura M, Zhao B, Honda Y (2006). Bone formation enhanced by implanted octacalcium phosphate involving conversion into Ca-deficient hydroxyapatite. Biomaterials.

[28] Kawai T, Echigo S, Matsui K, Tanuma Y, Takahashi T, Suzuki O (2014). First clinical application of octacalcium phosphate collagen composite in human bone defect. Tissue Eng Part A.

[29] Miura KI, Sasaki M, Ohba S, Noda S, Sumi M, Kamakura S (2022). Long-term clinical and radiographic evaluation after maxillary sinus floor augmentation with octacalcium phosphate-collagen composite: a retrospective case series study. J Tissue Eng Regen Med.

[30] Kawai T, Kamakura S, Matsui K, Fukuda M, Takano H, Iino M (2020). Clinical study of octacalcium phosphate and collagen composite in oral and maxillofacial surgery. J Tissue Eng.

